# Mitochondrial DNA sequence divergence and diversity of *Glossina fuscipes fuscipes* in the Lake Victoria basin of Uganda: implications for control

**DOI:** 10.1186/s13071-015-0984-1

**Published:** 2015-07-22

**Authors:** Agapitus B. Kato, Chaz Hyseni, Loyce M. Okedi, Johnson O. Ouma, Serap Aksoy, Adalgisa Caccone, Charles Masembe

**Affiliations:** Department of Biological Sciences, College of Natural Sciences, Makerere University, Box 7062, Kampala, Uganda; Department of Biology, University of Mississippi, Oxford, MS 38677 USA; National Livestock Resources Research Institute, Tororo, Uganda; Biotechnology Research Institute, Kenya Agricultural and Livestock Research Organization, Kikuyu, Kenya; Division of Epidemiology of Microbial Diseases, Yale School of Public Health, Yale University, New Haven, CT 06520 USA; Department of Ecology and Evolutionary Biology, Yale University, New Haven, CT 06520 USA

**Keywords:** *Glossina fuscipes fuscipes*, Tsetse fly, Trypanosomiasis, Vector control, Gene flow, Demographic dynamics

## Abstract

**Background:**

*Glossina fuscipes fuscipes* is the main vector of African Trypanosomiasis affecting both humans and livestock in Uganda. The human disease (sleeping sickness) manifests itself in two forms: acute and chronic. The Lake Victoria basin in Uganda has the acute form and a history of tsetse re-emergence despite concerted efforts to control tsetse. The government of Uganda has targeted the basin for tsetse eradication. To provide empirical data for this initiative, we screened tsetse flies from the basin for genetic variation at the mitochondrial DNA cytochrome oxidase II (mtDNA COII) gene with the goal of investigating genetic diversity and gene flow among tsetse, tsetse demographic history; and compare these results with results from a previous study based on microsatellite loci data in the same area.

**Methods:**

We collected 429 *Gff* tsetse fly samples from 14 localities in the entire Ugandan portion of the Lake Victoria coast, covering 40,000 km^2^. We performed genetic analyses on them and added data collected for 56 *Gff* individuals from 4 additional sampling sites in the basin. The 529pb partial mitochondrial DNA cytochrome oxidase II (mtDNA COII) sequences totaling 485 were analysed for genetic differentiation, structuring and demographic history. The results were compared with findings from a previous study based on microsatellite loci data from the basin.

**Results:**

The differences within sampling sites explained a significant proportion of the genetic variation. We found three very closely related mtDNA population clusters, which co-occurred in multiple sites. Although *Φ*_*ST*_ (0 – 0.592; P < 0.05) and Bayesian analyses suggest some level of weak genetic differentiation, there is no correlation between genetic divergence and geographic distance (r = 0.109, P = 0.185), and demographic tests provide evidence of locality-based demographic history.

**Conclusion:**

The mtDNA data analysed here complement inferences made in a previous study based on microsatellite data. Given the differences in mutation rates, mtDNA afforded a look further back in time than microsatellites and revealed that *Gff* populations were more connected in the past. Microsatellite data revealed more genetic structuring than mtDNA. The differences in connectedness and structuring over time could be related to vector control efforts. Tsetse re-emergence after control interventions may be due to re-invasions from outside the treated areas, which emphasizes the need for an integrated area-wide tsetse eradication strategy for sustainable removal of the tsetse and trypanosomiasis problem from this area.

**Electronic supplementary material:**

The online version of this article (doi:10.1186/s13071-015-0984-1) contains supplementary material, which is available to authorized users.

## Background

Tsetse flies (Diptera: Glossinidae) are the major vectors of Human African Trypanosomiasis (HAT) and Animal African Trypanosomoses (AAT) in sub-Saharan Africa [1, 2]. Approximately 70 million people in 1.55 million km^2^ are estimated to be at risk of HAT caused by two species of trypanosomes [3]: *Trypanosoma brucei gambiense* (*Tbg*), responsible for the chronic form of the disease, and *Trypanosoma brucei rhodesiense* (*Tbr*), which causes the acute form [4, 5]. There is evidence that tsetse have influenced food production, urbanization, and institutional development dating back to historical Africa [6]. AAT is a major obstacle to the development of more efficient and sustainable livestock production systems, and thus one of the most important causes of hunger and poverty [7, 8]. There are currently no vaccines for the above diseases, and the available drugs are expensive, toxic, and logistically difficult to administer.

Since reducing host/vector contact can rapidly slow human trypanosomiasis transmission [9], controlling the tsetse fly remains the most efficient and sustainable way of managing African trypanosomiasis. Available environmentally-friendly tsetse control techniques include the sequential aerosol technique (SAT), which is an aerial application of ultra-low-volume non-residual insecticides [10], the use of insecticide-impregnated targets and traps that can be odour-baited [11], the application of residual insecticides on livestock, referred to as the live bait technique [12], and the sterile insect technique (SIT) [13].

In 2001, the African Union established the Pan African Tsetse and Trypanosomiasis Eradication Campaign (PATTEC) with a view of using an integrated area-wide approach to control HAT and AAT with the available methods. A prerequisite to any vector control campaign aiming at eradication is to identify and target isolated populations to minimize the risk of reinvasion. If not already isolated, populations could be isolated by creating physical obstacles, such as the insecticide-impregnated biconical trap barriers. Such a method has been used to effectively control *Glossina palpalis gambiensis* and *Glossina tachinoides* in a 3000 km^2^ area in an agro-pastoral zone of Sideradougou, in the Guinea savannah in Cameroon [14].

Population genetic techniques can help understand and quantify gene flow between populations, which can be used as a proxy for dispersal [15]. Dispersal rates for *Glossina fuscipes fuscipes* (*Gff*) based on mark-release-recapture (MRR) studies are about 14.2 km per generation, given a movement estimate of 338 m/day [9].

Fine-scale genetic analysis based on microsatellites confirmed that *Gff* disperse up to 14 km per generation [16], *Gff* appear to be genetically homogeneous over 1–5 km^2^.

Information about dispersal derived using population genetic techniques can be used to support vector control decision-making [17, 18] at various spatial levels and ecological settings. For example, regional studies such as the one on riverine *Glossina palpalis palpalis* in west and central Africa [19] have provided information that is useful for control of riverine *palpalis* tsetse group in cross-boundary projects. Studies of tsetse in Burkina Faso, Guinea and Senegal have identified riverine tsetse populations that are sufficiently isolated to warrant attempts at complete eradication [20, 21]. In the *morsitans* or savannah tsetse group, population genetic studies have indicated high gene flow among *Glossina morsitans morsitans* populations separated at geographic scales of 12–917 km in East and Southern Africa [22, 23].

In Uganda, *Gff*, a riverine subspecies in the *palpalis* group, is the major vector of HAT. The acute form of HAT (*T. b. rhodesiense*) previously had its historical focus along the shores of Lake Victoria, but has recently extended its range northwards into central Uganda [4, 24]. If this distribution continues extending, the range might overlap with that of the chronic form of HAT (*T. b. gambiense*) found in northwestern Uganda, thereby complicating diagnosis, treatment, and providing new challenges, as recombination between the two trypanosome forms can occur and could lead to unforeseen pathologies [25, 26].

In an effort to eliminate the acute form of the disease and to prevent potential challenges associated with overlap of the two forms of HAT in Uganda, in 2008 Pan African Tsetse and Trypanosomiasis Eradication Campaign (PATTEC) activities were initiated against *Gff* in the Lake Victoria basin; an area with a history of tsetse re-emergence despite concerted tsetse control efforts [27]. Tsetse re-emergence is a major obstacle to elimination of the tsetse fly vector in Africa [28]. Understanding the population genetics of *Gff* in the Lake Victoria basin may elucidate the factors influencing re-emergence. Indeed, genetic tools have revealed genetic structuring among localities north, south and west of Lake Kyoga in Uganda, occurrence of gene flow among genetic clusters [29], and temporal stability of these genetic patterns [30]. We previously screened for genetic variation at 15 microsatellite loci using tsetse flies from 14 sampling sites from continental and island locations along Lake Victoria in Uganda [16]. That study identified four genetically distinct clusters and showed that gene flow occurred at varying levels between these clusters.

**Fig. 1:**
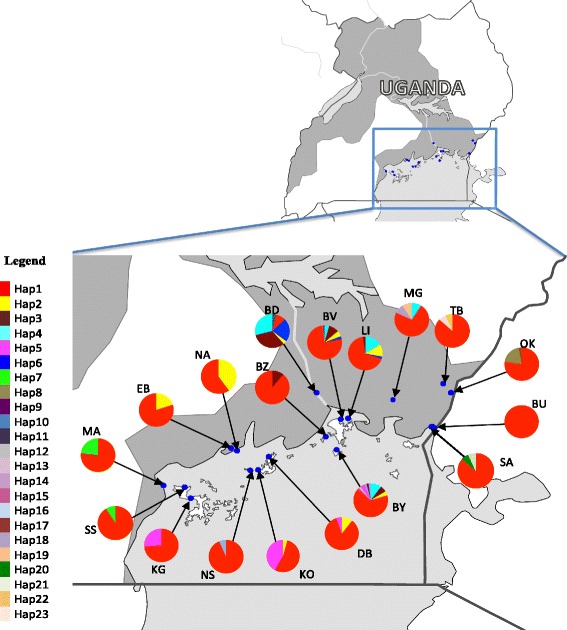
Map showing the location of the 18 sampling sites and the distribution of the 23 COII mtDNA haplotypes of *G. f. fuscipes* recovered from the analysis of 485 individuals in the Lake Victoria Basin, Uganda. Blue dots represent sampled localities, pie charts indicate frequencies of the haplotypes in the sampled localities and each colour in a pie chart represents a haplotype. The inset in the upper right corner shows the location of sampling sites with reference to the whole of Uganda and neighboring countries

In this study, we followed up on the work of our group [16] by screening 485 tsetse flies from 18 sampling sites (Fig. 1) for genetic variation in a fragment of the mtDNA COII gene (526 bp). In contrast to the bi-parentally inherited microsatellites, mtDNA is maternally inherited and lacks recombination [31, 32]. Given these differences, as well as the slower mutation rate in mtDNA than microsatellites [33, 34], we can compare differences in genetic variation among different timescales. The insight about temporal dynamics that the comparison of mtDNA and microsatellite data affords, could further inform the ongoing PATTEC control and monitoring efforts in the area and possibly beyond.

## Methods

### Sampling

We obtained 429 *Gff* tsetse fly samples from 14 localities in the entire Ugandan portion of the Lake Victoria coast, covering 40,000 km^2^ (Fig. 1). The samples were collected from continental (Masaka, MA; Entebbe, EB; Budondo, BD; Okame, OK and Busime, BU) and offshore islands on Lake Victoria (Buvuma Islands: Buvuma, BV; Bugaya, BY; Buziri, BZ and Lingira, LI; Koome islands: Damba, DB; Nsazi, NS and Koome, KO; Ssese islands: Kalangala, KG and Ssese, SS) as shown in Table 1a. A maximum of 30 individuals per location were collected between October 2009 and March 2011, using biconical traps [35], following standard protocols. Whole tsetse samples were stored individually in 90 % ethanol and kept at 4 °C.

**Table 1:** Sampling localities and genetic diversity statistics for the mitochondrial COII sequences from 18 *Gff* localities in the lake Victoria basin in Uganda*.* N = Number of individuals analyzed, Nh = Number of haplotypes, Hd = haplotype diversity and π = nucleotide diversity. (1a) New sampling localities for this study. (1b) Sampling localities added from previous studies (Echodu et al. 2013)

Location	Code	Longitudes	Latitudes	N	Nh	Hd	π
Table 1a							
Masaka	MA	31.9852	−0.2143	26	2	0.369	0.001
Ssese Island	SS	32.1883	−0.3167	11	2	0.182	0.000
Kalangala Island	KG	32.1437	−0.2283	23	2	0.403	0.008
Koome Island	KO	32.6879	−0.0911	24	3	0.554	0.001
Damba Island	DB	32.7659	0.0127	19	3	0.292	0.003
Nsazi Island	NS	32.6296	0.0956	15	2	0.133	0.001
Entebbe	EB	32.4852	0.0823	35	2	0.329	0.001
Buvuma Island	BV	33.2788	0.1368	73	7	0.423	0.002
Bugaya Island	BY	33.2684	0.0675	49	10	0.774	0.004
Buziri Island	BZ	33.1883	0.1716	9	2	0.222	0.001
Lingira Island	LI	33.3532	0.3168	43	6	0.527	0.002
Budondo	BD	33.1209	0.5208	31	6	0.565	0.004
Okame	OK	33.3532	0.3168	35	3	0.375	0.001
Busime	BU	33.9711	0.2508	36	1	0.000	0.000
Table 1b							
Nkumba	NA	32.5300	0.0600	15	2	0.514	0.002
Mayuge	MG	33.45585	0.422183	11	3	0.491	0.005
Sangalo	SA	33.99286	0.24204	15	3	0.257	0.001
Tuba	TB	34.05864	0.59159	15	3	0.257	0.001

### DNA extraction, Amplification and Sequencing

Total genomic DNA was extracted from legs of individual tsetse flies using the PrepGEM™ Insect kit (ZYGEM Corp. Ltd) as per the manufacturer’s protocol. A 570 bp fragment of mtDNA COII gene was PCR-amplified using the primers COIF1 (5’ – CCT CAA CAC TTT TTA GGT TTA G – 3’) and COIIR1 (5’ – GGT TCT CTA ATT TCA TCA AGT A – 3’), as described by [29]. Reactions contained 1–10 ng of template DNA, 2.6 μl (5X) buffer (GoTaq colorless, Promega), 1.1 μl (10 mM) dNTPs, 0.5 μl (10 mM) primers, 1.1 μl (25 mM) MgCl2, and 0.1 μl (U/μL) GoTaq polymerase, and 6.9 μl of water for a total volume of 13 μl. Amplification involved an initial denaturation step at 95 °C for 5 min, followed by 95 °C for 30 s of denaturation, 40 cycles each for 30 s at 50 °C for annealing, 45 s at 72 °C for extension and a final extension step at 72 °C for 20 min. The PCR products were purified using ExoSAP-IT (Affymetrix, Inc.) as per the manufacturer’s protocol. Sequencing was carried out for both forward and reverse strands at the DNA Analysis Facility on Science Hill at Yale University (http://dna-analysis.research.yale.edu/).

Chromatograms were visually inspected and sequences trimmed to remove poor quality data using the CLC Workbench (CLC Bio Denmark). The forward and reverse strands were used to create a consensus sequence for each sample. In addition to the newly sequenced 429 samples, mtDNA COII gene sequences for 56 *Gff* individuals from 4 additional sampling sites in the basin [36] (Table 1b) were added to the dataset. Thus, making the final number of analysed sequences from the same sampling sites where previous microsatellite data were collected [16] 485. The total length of these sequences, prior to analysis, was 570 bp. This fragment was trimmed to a 526 bp long fragment common to all the samples.

### Genetic diversity, network and population structure analysis

We analyzed the data for haplotype diversity (Hd) and nucleotide diversity (π) using DnaSP version 5.10 [37]. Significance was assessed with 1000 permutations. The partitioning of the genetic diversity within and among sampling sites was evaluated using the analysis of molecular variance (AMOVA) as implemented in Arlequin 3.5 [38]. We performed a nested analysis of variance (AMOVA) framework to partition the total amount of genetic differentiation between hierarchical levels of population subdivision [39], and produced Φ-statistics that measure the similarity of pairs of haplotypes in each hierarchical level of the analysis, relative to pairs drawn from the pool of sequences in the higher hierarchical level. Significance of the Φ-statistics was tested by permuting haplotypes among the corresponding hierarchical levels, and recalculating the statistics to obtain their null distributions [40].

To understand the evolutionary relationship of the mtDNA haplotypes, we constructed a median-joining haplotype network [41], where individual sequences were collapsed into haplotypes using the default settings in the NETWORK 4.6.1 software (http://fluxus-engineering.com). This program implements the median-joining method in the absence of recombination. The method, which provides an estimation of the haplotype genealogical relationships, is a more powerful method than bifurcating trees, when studying phylogenetic relationships at the intraspecific level, because it allows for the inclusion of multi-furcations and reticulations [42]. The program GenGIS [43] was used to visualize haplotype diversity and its relationship between geographical localities.

Genetic differentiation among the 18 sampling sites was evaluated with and without spatial information as a priori [44], using the Bayesian approach implemented in BAPS 6 [45]. We employed the spatial model option in BAPS, using local populations inhabiting discrete habitat patches (localities) with known geographical coordinates as the population units to be clustered. All molecular data collected from a particular local population were used to obtain the posterior distribution of haplotype frequencies for that population. Under the spatial model, the genetic structure is calculated assuming *a priori* that the structure within a particular area depends on the neighbouring areas. This program uses a statistical genetic model that treats nucleotide frequencies and K (the number of genetically diverged groups in a population) as random variables. The best K was determined using posterior probabilities. The best partition was visualized using a Voronoi tessellation as implemented in BAPS.

To obtain pairwise estimates of genetic differentiation we computed *Φ*_*ST*_ values among sampling sites using Arlequin 3.5 with 1000 random permutations. We used *Φ*_*ST*_ because it also accounts for the evolutionary relatedness of the mtDNA haplotypes. To test the correlation between these pairwise genetic distances and pairwise geographic distances, we used Mantel’s test [46] with 9,999 permutations, as implemented in GenAlEx 6.5. Pairwise geographic (Euclidean) distances were generated using the coordinates of the sampling localities in GenAlEx 6.5 [47].

### Demographic history

We used mismatch distributions (number of pairwise mutational differences) [48] to determine if the mtDNA data showed signatures of population expansion and calculated the raggedness statistic to analyse the goodness of fit of the population expansion model to evaluate the extent to which the distribution followed the smooth unimodal curve, which one would expect under a population growth scenario. However, as this approach does not use all the information in the sequence data, we also used Tajima’s *D* [49] and Fu’s *F*_*S*_ [50] statistics to test for deviations from neutral expectations. Positive values indicate an excess of intermediate-frequency haplotypes, which might result from balancing selection or bottlenecks, while negative values reflect an excess of rare polymorphisms, which might result from population growth but also genetic hitchhiking, selective sweeps, or background selection. For all these tests we used DnaSP version 5.10 [37] and significance was evaluated by comparing observed and expected statistics to a distribution of values generated with 5000 coalescent simulations.

## Results

### Genetic diversity

A 526 bp fragment of the mtDNA cytochrome oxidase II (COII) was analysed from 485 individuals from 18 localities around the Lake Victoria basin (Table 1, Fig. 1). The collection of sequences was comprised of 23 haplotypes and 29 polymorphic sites (Table 2). The number of haplotypes within each sampling site varied considerably (from 1 to 10 haplotypes per sampling location) despite equal sample sizes. Similarly, both haplotype diversities ranged widely from 0 in BU to 0.774 in BY (Table 1). On the contrary, nucleotide diversity was very low ranging from 0 in BU a coastal site and SS an island site, to 0.008 in KG in Ssese islands. These low levels of nucleotide diversity may be due to relatively recent reduction in population size or recent colonization events, as sampling effort was the same for every site (Table 1). However, the fact that for some sites we recovered high haplotypic diversity suggests differences in demographic dynamics among sites.

**Table 2:** Haplotype distributions among the 18 *G. f. fuscipes* studied, based on mitochondrial CO II sequence data: 1^st^ column: Haplotype code name (Hap1-Hap23); 2^nd^ column: segregating sites in each haplotype, numbers on top of 2^nd^ column are the variable sites in the reference sequence JFJR01006635.1, dots represent identical nucleotides to the ones for Hap1. The location code names (column 3 to 20) are those shown in Table 1. The last column shows the frequency of each haplotype in the whole mitochondrial CO II sequence data

Haplotype	Segregating sites	Localities																	
		MA	SS	KG	KO	DB	NA	NS	EB	BV	BY	BZ	LI	BD	MG	BU	OK	SA	TB
Hap1		20	10	17	13	16	9	14	28	55	32	8	29	3	8	36	27	13	13
Hap2		-	-	-	1	2	6	-	7	4	2	-	5	1	-	-	-	-	-
Hap3		-	-	-	-	-	-	-	-	3	5	-	5	9	-	-	-	-	-
Hap4		-	-	-	-	-	-	-	-	7	3	1	2	10	-	-	-	-	-
Hap5		-	-	6	10	1	-	-	-	-	2	-	-	-	-	-	-	-	-
Hap6		-	-	-	-	-	-	-	-	2	-	-	1	7	-	-	-	-	-
Hap7		6	1	-	-	-	-	-	-	-	-	-	1	-	-	-	-	-	-
Hap8		-	-	-	-	-	-	-	-	-	-	-	-	-	-	-	7	-	-
Hap9		-	-	-	-	-	-	-	-	1	1	-	-	-	-	-	-	-	-
Hap10		-	-	-	-	-	-	-	-	-	-	-	-	1	-	-	-	-	-
Hap11		-	-	-	-	-	-	-	-	1	-	-	-	-	-	-	-	-	-
Hap12		-	-	-	-	-	-	-	-	-	1	-	-	-	-	-	-	-	-
Hap13		-	-	-	-	-	-	-	-	-	1	-	-	-	-	-	-	-	-
Hap14		-	-	-	-	-	-	-	-	-	1	-	-	-	-	-	-	-	-
Hap15		-	-	-	-	-	-	-	-	-	1	-	-	-	-	-	-	-	-
Hap16		-	-	-	-	-	-	1	-	-	-	-	-	-	-	-	-	-	-
Hap17		-	-	-	-	-	-	-	-	-	-	-	-	-	-	-	1	-	-
Hap18		-	-	-	-	-	-	-	-	-	-	-	-	-	1	-	-	-	-
Hap19		-	-	-	-	-	-	-	-	-	-	-	-	-	1	-	-	-	-
Hap20		-	-	-	-	-	-	-	-	-	-	-	-	-	-	-	-	1	-
Hap21		-	-	-	-	-	-	-	-	-	-	-	-	-	-	-	-	1	-
Hap22		-	-	-	-	-	-	-	-	-	-	-	-	-	-	-	-	-	1
Hap23		-	-	-	-	-	-	-	-	-	-	-	-	-	-	-	-	-	1
Totals		26	11	23	24	19	15	15	35	73	49	9	43	31	11	36	35	15	15

**Fig. 2:**
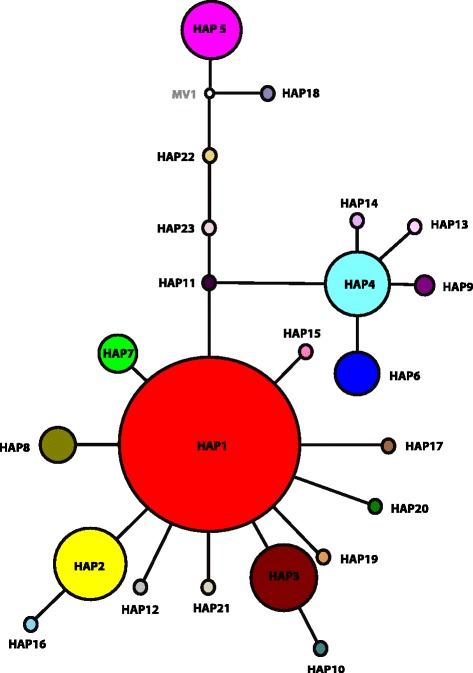
Median-Joining network [41] for 23 COII mtDNA haplotypes of *G. f. fuscipes* from 485 individuals in the Lake Victoria Basin, Uganda. Each colour represents a haplotype and the size of the circle is proportional to the number of individuals with that haplotype. Each line represents one mutational step, colour coding is the same as that in Fig. 1 and a white circle represents an inferred missing haplotype

Table 3 shows the results for the AMOVA analysis on the 18 sampling sites; overall genetic variation within sampling sites was much larger (85.21 %) than the variation among sampling sites (14.79 %), which is indicative of shallow levels of genetic divergence among sampling sites. This is further supported by the distribution of haplotypes among the sampled localities (Fig. 1, Tables 1 and 2) and their evolutionary relationships (Fig. 2).

**Table 3:** Results of AMOVA (Excoffier et al. 1992) on 485 mitochondrial COII sequences from 18 localities in the Lake Victoria Basin, Uganda, computed using the Arlequin program (Excoffier et al. 2009). Significance was tested using 1000 random permutations

Source of		Variance	Percentage
Variation	d.f.	Components	Of variation
Among			
Localities	17	0.03491 Va	14.79 %
Within			
Localities	467	0.20107 Vb	85.21 %

Figure 1 shows that Haplotype 1 (HAP1), the most common haplotype (72.4 %; Table 2), is ubiquitous. The second most common haplotype, HAP2 (Table 2) was by far much less frequent (3.9 %) than HAP1 and was found in only 8 localities. Six other haplotypes occurred in two or more localities. These eight haplotypes represented 95.7 % of the sample. The other fifteen haplotypes (4.3 % of the sample) were unique to specific localities. The high percentage of shared haplotypes with the most common haplotype found at all sampling sites suggests high connectivity of *Gff* in the past. However, some haplotypes were retrieved from only geographically proximate areas, suggesting the occurrence of some genetic structuring. For example, HAP4 (Table 2; Light-blue in Fig. 1) was retrieved from BD, BV, LI, BZ, MG and BY, all geographically proximate localities; HAP7 (Green in Fig. 1) appears only in the extreme west of the basin (SS and MA), and HAP8 was retrieved exclusively from OK, a sampling site at the eastern edge of the *Gff* belt (Fig. 1; Table 2). Interestingly, HAP5 occurred exclusively on islands, particularly sites KG, NS, KO, DB and BY, some of which are located more than 100 km apart.

Figure 2 shows the evolutionary relationships among the 23 haplotypes. The network shows two haplogroups separated by five mutational steps. The most common haplotype (HAP1) is located internally in the larger haplogroup, with the other haplotypes arising from it, suggesting that HAP1 is the ancestral haplotype of this haplogroup. In addition, a star-like polytomy separated from HAP1 by two mutation steps was found in this haplogroup. The second haplogroup has only two haplotypes, HAP5 and HAP18, each separated by one mutation step from an unknown haplotype. Overall the network shows very low levels of sequence divergence among haplotypes and a high frequency of singletons (i.e., haplotypes seen only once in a group of samples), a pattern suggesting recent divergence and possibly population expansion.

### Demographic history

To investigate demographic history and explore evidence of recent population expansions or reductions, we carried out mismatch distribution analyses by combining all the sampling sites (Additional file 1: Figure S1). Harpending’s Raggedness index rejected the null hypothesis of exponential growth (r > 0.05, P > 1.000). The observed distributions suggest a unimodal pattern, indicating a signal of past population expansion. Tajima’s *D* and Fu’s *F*_*S*_ (Table 4) were both negative and significant for the study area (*D* = −1.661; P = 0.014; *F*_*S*_ = −10.787, P = 0.009), confirming population expansion of the *Gff* population in this part of the basin. At locality level, however, *F*_*S*_ and *D* statistics (Table 4) confirmed demographic dynamics being different among localities as the values were negative for some sites and positive for the others.

**Table 4:** Neutrality and Demographic parameters: Tajima’s D, Fu’s Fs, Harpending’s raggedness index (r) based on mitochondrial COII sequence data of 18 localities of *G. f. fuscipes* belonging to the Lake Victoria Basin as implemented in the program DnaSP (Librado and Rozas 2009) for population size changes. In bold are statistically significant values at 0.05 Significance level

Locality	Tajima's D	Fu’s Fs	Ragged-ness Index (r)
MA	0.669; P = 0.839	1.003; P = 0.568	0.205; P = 0.200
SS	−1.129; P = 0.156	−0.410; P = 0.150	0.438; P = 0.490
KG	1.645; P = 0.950	9.668; P = 0.999	0.681; P = 0.360
KO	2.163; P = 0.989	8.861; P = 0.998	0.649; P = 0.910
DB	**−2.102; P = 0.005**	2.120; P = 0.880	0.589; P = 0.560
NA	1.769; P = 0.976	2.710; P = 0.887	0.765; P = 0.050
NS	**−1.685; P = 0.012**	0.834; P = 0.479	0.787; P = 0.750
EB	0.698; P = 0.792	2.352; P = 0.855	0.667; P = 0.000
BV	−1.104; P = 0.133	−2.334; P = 0.098	0.174; P = 1.000
BY	**−1.800; P = 0.017**	−2.325; P = 0.144	0.135; P = 0.580
BZ	−1.088; P = 0.209	−0.263; P = 0.173	0.358; P = 0.310
LI	−0.879; P = 0.207	−0.928; P = 0.298	0.255; P = 0.260
MG	**−1.903; P = 0.015**	1.313; P = 0.763	0.197; P = 1.000
BU	0.000; P = 1.000	0.000; P = N.A.	0.000; P = 0.000
OK	−0.400; P = 0.360	−0.341; P = 0.294	0.192; P = 0.350
SA	−1.491; P = 0.064	**−1.546; P = 0.022**	0.302; P = 0.460
TB	−0.948; P = 0.222	−0.006; P = 0.379	0.566; P = 0.530
Study Area	**−1.661; P = 0.014**	**−10.787; P = 0.009**	0.179; P = 1.000

### Population differentiation patterns

**Fig. 3:**
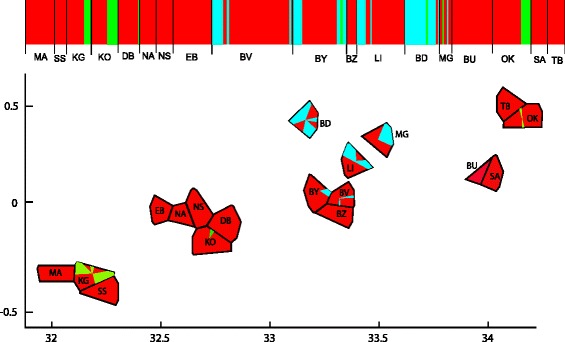
Genetic clustering of local populations in the Lake Victoria basin inferred with the program BAPS [44] using mtDNA COII marker. Locality codes are those described in Table 1 (**a**) Mixture clustering graphical output for K = 3, where K is the optimal number of clusters identified. Each vertical block is a sampling site, colour indicates membership of its individuals to population clusters (red - cluster 1, blue – cluster 2, green – cluster 3). Localities are ordered geographically from west to east across the basin. **b** Spatial clustering model for K = 3, each bordered cell represents a sampling site and colour indicates membership of its individuals to the same three population clusters as in A. X and Y-axes are spatial coordinates of the localities

Figure 3 shows results of the BAPS analyses. The analysis, which incorporates spatial information of sampling sites as prior information inferred existence of three (K = 3) genetic clusters. In agreement with the shallow genetic divergence and haplotypic distribution shown above, these clusters do not group entirely according to geographical location of tsetse samples. For instance, cluster 1 (red in Fig. 3), the cluster that groups the majority of individuals (67.8 %) includes tsetse flies from all sampling sites regardless of their geographic proximity. On the other hand there is some evidence of genetic structuring, because cluster 2 (blue in Fig. 3) includes only individuals from each of the Buvuma archipelago sites (LI, BY, BV, BZ) as well as samples from BD, a mainland site about 50 km away from the Buvuma islands. However, cluster 3 (green in Fig. 3) includes individuals from two different island groups (KG and KO) on the west side of the study area and OK, located at the opposite end of the *Gff* distribution in the Lake Victoria basin.

Similar conclusions in terms of overall levels of genetic divergence can be inferred from the pairwise *Φ*_*ST*_ values (Table 5). Among localities these values ranged from zero between populations from Damba Island (DB) and Nsazi island (NS), located about 4 km apart in the Koome archipelago, to relatively high and statistically significant values between Budondo (BD) and Busime (BU; *Φ*_*ST*_ = 0.592, P ≤ 0.05), which are continental sites about 100 km apart. Samples from Lingira (LI), an island site in Buvuma islands and Nkumba (NA), a continental site about 40 km away, were not genetically distinct (*Φ*_*ST*_ = 0.05, P < 0.05), suggesting that there has been gene flow between islands and continental sampling sites. Surprisingly, samples from KO, a site only 5 km from DB and NS in the Koome archipelago, were genetically distinct from all samples including those from DB and NS which are only 5 km within the same archipelago, but similar to samples from KG, an island site more than 100 km away in Kalangala islands, suggesting possible long-range dispersal among the islands’ *Gff*. Additional file 2: Figure S2 shows the results of the Mantel test, which suggests no correlation between genetic and geographic distances among localities (*R* =0.109, P = 0.185), confirming the findings from the *Φ*_*ST*_ and the BAPS analyses.

**Table 5:** Pairwise differentiation estimates of mtDNA Φst between the 18 localities arranged from West to East across the basin: Computed in Arlequin 3.5 (Excoffier et al. 2009), bold numbers show statistically significant comparisons at 0.05 Significance level

	WEST									EAST									
WEST		MA	SS	KG	KO	DB	NA	NS	EB	BV	BY	BZ	LI	BD	MG	BU	OK	SA	TB
	MA	-																	
	SS	−0.003	-																
	KG	**0.105**	**0.083**	-															
	KO	**0.21**	**0.228**	0.024	-														
	DB	**0.058**	−0.02	0.042	**0.174**	-													
	NA	**0.19**	**0.214**	**0.182**	**0.186**	**0.116**	-												
	NS	**0.088**	−0.037	**0.115**	**0.269**	0	**0.264**	-											
	EB	**0.093**	0.04	**0.107**	**0.219**	−0.02	**0.054**	**0.063**	-										
EAST	BV	**0.054**	0.008	**0.066**	**0.184**	−0.001	**0.12**	0.028	0.023	-									
	BY	**0.054**	0.036	0.038	**0.107**	0.02	**0.084**	**0.062**	0.043	0.003	-								
	BZ	0.043	−0.051	**0.063**	**0.202**	−0.03	**0.183**	−0.033	0.027	−0.033	0.006	-							
	LI	**0.053**	0.038	**0.068**	**0.143**	0.014	**0.05**	**0.069**	0.019	0.002	−0.012	0.014	-						
	BD	**0.374**	**0.399**	**0.354**	**0.293**	**0.388**	**0.288**	**0.438**	**0.404**	**0.348**	**0.252**	**0.352**	**0.265**	-					
	MG	0.033	0.007	0.039	**0.133**	0	**0.107**	0.046	0.035	−0.014	−0.028	−0.016	−0.022	**0.272**	-				
	BU	**0.238**	**0.127**	**0.287**	**0.457**	**0.134**	**0.51**	**0.065**	**0.179**	**0.101**	**0.16**	**0.186**	**0.175**	**0.592**	**0.283**	-			
	OK	**0.085**	0.041	**0.096**	**0.21**	0.044	**0.187**	**0.065**	**0.08**	0.046	0.049	0.026	**0.058**	**0.385**	0.024	**0.183**	-		
	SA	**0.053**	−0.038	**0.074**	**0.215**	−0.018	**0.196**	−0.022	0.037	0.006	0.03	−0.047	0.036	**0.391**	−0.004	**0.118**	0.037	-	
	TB	**0.053**	−0.038	**0.074**	**0.215**	−0.018	**0.196**	−0.022	0.037	0.006	0.03	−0.047	0.036	**0.391**	−0.004	**0.118**	0.037	−0.033	-
	WEST									EAST									

## Discussion

### Lack of mtDNA structure in Lake Victoria basin Gff

Sequence analysis of the COII mitochondrial DNA fragment from *Gff* populations across the Lake Victoria basin revealed very little genetic structuring. Most of the genetic variation at this locus was found within rather than between sampling sites (Table 3). Bayesian clustering inferred three spatially overlapping clusters, which do not group according to geographical origin of the samples. The overlapping spatial clustering could be a result of stochasticity in the process of lineage sorting of haplotypes followed by introgression due to gene flow from continental sites not included in this study, resulting in spatial mixing of the haplotype groups. A previous study indeed showed high levels of gene flow among different continental sampling sites separated by hundreds of kilometers in both Southern and Northern Uganda [29], reinforcing this hypothesis. Given the data at hand, it is not possible to distinguish between ancestral polymorphisms or recent introgression, as both could produce the observed patterns [51]. On the other hand the influence of reproductively inherited symbionts such as *Wolbachia* [52] could be investigated.

Regardless of the very little genetic structuring that we detected among sampling sites, we found relatively high levels of genetic diversity, as 14 of the 23 haplotypes recovered in this study are singletons (Table 2). Although this could reflect technical artifacts rather than the actual diversity of this mtDNA fragment, we feel that this is unlikely for a variety of reasons. The observed mtDNA sequence diversity is unlikely to be due to the presence of transcriptionally inactive mtDNA fragments inserted in the nuclear genome, numts [53]. We did not find evidence of mixed templates when sequencing the PCR products, or stop codons when the DNA sequences were translated into amino acids. Moreover, numts were never observed in any of previous studies of *Gff* mtDNA polymorphism, which included samples from a larger spatial scale than the current study [29, 30, 36, 54]. It is therefore unlikely that the patterns observed in the mtDNA data could be attributed to accidental cross-contamination or sample mixing, given that we checked for cross-contamination at each step, including negative controls. Indeed data were collected for both markers at the same time from the same DNA extractions and the microsatellite markers did not show any evidence of cross-contamination [16]. Additionally, several samples were genotyped and sequenced in duplicate and yielded identical results.

### Genetic drift and gene flow equilibrium in Lake Victoria basin Gff

The Mantel test (Additional file 2: Figure S2) detected no significant correlation between geographic and genetic distance, and pairwise *Φ*_*ST*_ comparisons showed higher differentiation between geographically close localities than distant localities, which suggests the existence of a complex and locality-dependent population. This could be facilitated by local environmental conditions, which would allow both genetic drift and gene flow to occur concurrently. *Gff* are found in highly fragmented habitats where genetic drift could be the predominant force. However, *Gff* also occur in contiguous riverine habitats along Lake Victoria and the Nile River, which can facilitate gene flow by acting as a corridor for individual dispersal among localities across the basin. The role of contiguous riverine habitat in facilitating long-range dispersal in tsetse has been previously discussed for the same species in Uganda but at a larger geographic scale [36, 55] and also for another riverine tsetse species *G. tachinoides* in Burkina Faso [56].

The haplotype network depicts a frequent haplotype (HAP1), with the majority of the haplotypes (91.3 %) in the network originating from it. This haplotype has a range-wide distribution across the basin, and more than 95 % of all the haplotypes are shared among the localities, suggesting long-range gene flow across the basin. Despite the long-range gene flow, some haplogroups were retrieved from only geographically proximate localities, which, coupled with the presence of private haplotypes at some localities further supports the importance of both gene flow and genetic drift in shaping the observed genetic patterns. Localities around the source of the Nile, such as BD, BV, LI, BY and MG (Fig. 1) had the highest haplotype diversities, confirming the role of contiguous riverine habitats in facilitating gene flow and the importance of the river Nile in facilitating gene flow between the lake Victoria basin and the northern *Gff* lineage, as previously suggested [29]. Interestingly, one haplogroup was exclusively retrieved from islands, some of which are more than 100 km apart. This haplogroup is five mutation steps from the dominant haplogroup, indicating higher connectivity among the *Gff* populations across islands than to the coastal area, suggesting that the islands could have been connected in the past.

### Localized demographic dynamics

Both mismatch distributions and neutrality tests indicated demographic expansion for the study area, but the difference in demographic dynamics exhibited by the neutrality tests at locality level is further evidence for population sub-division rather than panmixia of *Gff* in the basin. The positive *Fs* and *D* values indicate that *Gff* experienced localized population reductions or re-colonization events at some localities as opposed to the expansions at the other localities showing negative values. This could be a result of unsustained small-scale tsetse control projects that register temporally successes at those localities, but are followed by re-infestations from adjacent un-treated areas when the projects end.

### Comparison between mitochondrial and nuclear DNA markers

In another study of *Gff* [16] in the Lake Victoria basin, frequency-based analysis of microsatellite genotypic data revealed a complex genetic structure with four distinct meta-populations, which, although genetically distinct and spatially separated, also showed considerable amounts of gene flow. The microsatellite analyses also revealed existence of isolation by distance (IBD) within and between the distinct genetic clusters. Genetically derived dispersal distances varied between clusters ranging from about 2.5 to 14 km and matched reasonably well with dispersal rates predicted from mark–release–recapture (MRR) data for *Gff* and other riverine species [9]. Hierarchical *F*_*ST*_ and individual assignment tests indicated that there were four genetic clusters, and that flies in clusters 3 and 4 shared many migrants, while clusters 1 and 2 were more isolated. The difference in gene flow among these clusters was attributed to heterogeneity in human influence. Clustering of *Gff* from island sites with *Gff* from mainland sites led to a conclusion that the Lake Victoria does not act as a barrier to fly movement and gene flow, possibly due to passive dispersal mediated by boat traffic.

The results presented in this study show both agreements and disagreements with previous results [16]. Both studies recorded high gene flow between islands and adjacent mainland sites; however, they differed in the level of genetic structuring that was identified. Unlike mitochondrial DNA, microsatellite data indicated the presence of four distinct genetic clusters in a small area, with different degrees of isolation from the rest. Additionally, in contrast to mitochondrial DNA, which indicated population expansion throughout *Gff* demographic history, microsatellites pointed to population stability over several generations in the Lake Victoria region [16, 30] as well as other areas in Uganda [36]. Since mitochondrial DNA has lower mutation rates than microsatellites [33, 34], does not recombine [57], and has a smaller effective population size because of its maternal inheritance, it provides insights on older evolutionary events than microsatellite data [58]. So, by revealing patterns further back in the demographic history of *Gff* in the Lake Victoria region, the mtDNA results in this study complement inferences based on microsatellites [16]. One mtDNA haplotype was present in all sampling sites, suggesting a higher degree of connectivity between these sites in the past. It is possible that due to human activity, especially vector control efforts and human development, *Gff* populations have become more and more fragmented, which is why the microsatellites reveal more genetic structuring. The *Gff* structuring revealed by microsatellites in this study is also in line with recent work modeling predictions of *Gff* distributions in southern Uganda [59].

## Conclusion

Results of gene structuring and connectivity based on partial mtDNA sequences alone may underestimate current levels of genetic differentiation. As revealed by microsatellite data, lack of significant partitioning among groups or populations based on mtDNA data may not necessarily be indicative of current panmixia, but instead reflects historical events. This study has revealed the demographic history of *Gff* in the Lake Victoria basin, enabling us to better understand the factors behind the observed tsetse re-emergences after successful control interventions in the basin.

In terms of tsetse and trypanosomiasis control, interventions implemented at local scales are unlikely to produce long-lasting results due to re-invasion(s) from adjacent areas and/or residual tsetse pockets. As such, the high levels of genetic mixing between *Gff* in the island and mainland sites suggests that island and the mainland populations should be handled at the same time when implementing interventions. These findings support the need for an integrated area-wide elimination strategy for tsetse and trypanosomiasis from Uganda.

## Additional files

Additional file 1: Figure S1. Mismatch distributions plot [48] obtained using pairwise differences in mitochondrial COII sequence nucleotides for *Glossina f. fuscipes* in the lake Victoria Basin, Uganda. On the X-axis are the pairwise nucleotide differences, Y-axis are the number of pairs (Frequency). The solid grey lines show observed frequency distribution while the dotted black lines show the distribution expected under constant growth. The data were obtained using DNASP version 5.10 [37]. Additional file 2: Figure S2. Mantel Test plot of genetic distance (Φst /(1- Φst)) versus geographic distance for pairwise comparisons among 18 localities of *G. f. fuscipes* in the lake Victoria Basin, Uganda. Blue dots represent pairwise comparisons of localities and the black line is the linear correlation of genetic and geographic distances across the basin. There is no isolation by distance (R = 0.109, P value = 0.185).

## References

[1] Leak SGA. Tsetse biology and ecology: their role in the epidemiology and control of trypanosomosis. Wallingford: CABI; 1998.

[2] Van den Bossche P, de La Rocque S, Hendrickx G, Bouyer J (2010). A changing environment and the epidemiology of tsetse-transmitted livestock trypanosomiasis. Trends Parasitol.

[3] Simarro PP, Cecchi G, Franco JR, Paone M, Diarra A, Ruiz-Postigo JA, et al. Estimating and Mapping the Population at Risk of Sleeping Sickness. PLoS Negl Trop Dis. 2012. doi:10.1371/journal.pntd.0001859 10.1371/journal.pntd.0001859 PMC3493382 23145192

[4] Matovu E, Stewart ML, Geiser F, Brun R, Mäser P, Wallace LJ, Burchmore RJ, Enyaru JC, Barrett MP, Kaminsky R, Seebeck T, de Koning HP (2003). Mechanisms of arsenical and diamidine uptake and resistance in Trypanosoma brucei. Eukaryot Cell.

[5] Cecchi G, Paone M, Franco JR, Fèvre EM, Diarra A, Ruiz JA (2009). Towards the Atlas of human African trypanosomiasis. Int J Health Geogr.

[6] Alsan BM, Alesina A, Bates R, et al. The Effect of the TseTse Fly on African Development. American Economic Review. 2015;105(1):382–410.

[7] Cecchi G, Mattioli RC, Slingenbergh J, La Rocque SD, Feldmann U (2008). Standardizing land cover mapping for tsetse and trypanosomiasis decision making. PAAT Tech Sci Ser.

[8] Molyneux D, Hallaj Z, Keusch GT, McManus DP, Ngowi H, Cleaveland S, Ramos-Jimenez P, Gotuzzo E, Kar K, Sanchez A, Garba A, Carabin H, Bassili A, Chaignat CL, Meslin FX, Abushama HM, Willingham AL, Kioy D (2011). Zoonoses and marginalised infectious diseases of poverty: where do we stand?. Parasit Vectors.

[9] Rogers D (1977). Study of a Natural Population of Glossina fuscipes fuscipes Newstead and a Model of Fly Movement. J Anim Ecol.

[10] Jordan AM, Curtis CF (1972). Productivity of Glossina morsitans morsitans Westwood maintained in the laboratory, with particular reference to the sterile-insect release method. Bull World Health Organ.

[11] Green CH (1994). Advances in Parasitology Volume 34. Adv Parasitol.

[12] Thomson PC, Marlow NJ, Rose K, Kok NE (2000). The effectiveness of a large-scale baiting campaign and an evaluation of a buffer zone strategy for fox control. Wildl Res.

[13] Vreysen MJB, Saleh KM, Ali MY, Abdulla AM, Zhu ZR, Juma KG, Dyck VA, Msangi AR, Mkonyi PA, Feldmann HU (2000). Glossina austeni (Diptera: Glossinidae) Eradicated on the Island of Unguja, Zanzibar, Using the Sterile Insect Technique. J Econ Entomol.

[14] Mamoudou A, Zoli A, Delespaux V, Cuisance D, Geerts S, van den Bossche P (2009). Half a century of tsetse and animal trypanosomosis control on the Adamawa plateau in Cameroon. Rev Elev Med Vet Pays Trop.

[15] Gooding RH, Krafsur ES (2005). Tsetse genetics: contributions to biology, systematics, and control of tsetse flies. Annu Rev Entomol.

[16] Hyseni C, Kato AB, Okedi LM, Masembe C, Ouma JO, Aksoy S (2012). The population structure of Glossina fuscipes fuscipes in the Lake Victoria basin in Uganda: implications for vector control. Parasit Vectors.

[17] Solano P, Kaba D, Ravel S, Dyer NA, Sall B, Vreysen MJ, Seck MT, Darbyshir H, Gardes L, Donnelly MJ, De Meeûs T, Bouyer J (2010). Population genetics as a tool to select tsetse control strategies: Suppression or eradication of Glossina palpalis gambiensis in the niayes of senegal. PLoS Negl Trop Dis.

[18] Aksoy S, Caccone A, Galvani AP, Okedi LM (2013). Glossina fuscipes populations provide insights for human African trypanosomiasis transmission in Uganda. Trends Parasitol.

[19] Melachio TTT, Simo G, Ravel S, De Meeûs T, Causse S, Solano P (2011). Population genetics of Glossina palpalis palpalis from central African sleeping sickness foci. Parasit Vectors.

[20] Kagbadouno MS, Camara M, Bouyer J, Courtin F, Onikoyamou MF, Schofield CJ (2011). Progress towards the eradication of Tsetse from the Loos islands, Guinea. Parasit Vectors.

[21] Koné N, Bouyer J, Ravel S, Vreysen MJB, Domagni KT, Causse S (2011). Contrasting population structures of two vectors of African Trypanosomoses in Burkina Faso: Consequences for control. PLoS Negl Trop Dis.

[22] Ouma JO, Marquez JG, Krafsur ES (2007). Patterns of genetic diversity and differentiation in the tsetse fly Glossina morsitans morsitans Westwood populations in East and southern Africa. Genetica.

[23] Krafsur ES, Marquez JG, Ouma JO (2008). Structure of some East African Glossina fuscipes fuscipes populations. Med Vet Entomol.

[24] Waiswa C, Picozzi K, Katunguka-Rwakishaya E, Olaho-Mukani W, Musoke RA, Welburn SC (2006). Glossina fuscipes fuscipes in the trypanosomiasis endemic areas of south eastern Uganda: apparent density, trypanosome infection rates and host feeding preferences. Acta Trop.

[25] Hao Z, Kasumba I, Lehane MJ, Gibson WC, Kwon J, Aksoy S (2001). Tsetse immune responses and trypanosome transmission: implications for the development of tsetse-based strategies to reduce trypanosomiasis. Proc Natl Acad Sci U S A.

[26] Hamilton PB, Gibson WC, Stevens JR (2007). Patterns of co-evolution between trypanosomes and their hosts deduced from ribosomal RNA and protein-coding gene phylogenies. Mol Phylogenet Evol.

[27] Luyimbazi F: Detailed work plan/action plan for the collection of entomological baseline data. Integrated area-wide program for the creation of sustainable tsetse and trypanosomiasis free areas in the Lake Victoria basin. Entebbe: Ministry of Agriculture, Animal Industry and Fisheries; 2006.

[28] De La Rocque S, Augusseau X, Guillobez S, Michel V, De Wispelaere G, Bauer B (2001). The changing distribution of two riverine tsetse flies over 15 years in an increasingly cultivated area of Burkina Faso. Bull Entomol Res.

[29] Beadell JS, Hyseni C, Abila PP, Azabo R, Enyaru JCK, Ouma JO, Mohammed YO, Okedi LM, Aksoy S, Caccone A (2010) Phylogeography and population structure of Glossina fuscipes fuscipes in Uganda: Implications for control of tsetse. PLoS Negl Trop Dis. doi:10.1371/journal.pntd.0000636 10.1371/journal.pntd.0000636 PMC2838784 20300518

[30] Echodu R, Beadell JS, Okedi LM, Hyseni C, Aksoy S, Caccone A (2011). Temporal stability of Glossina fuscipes fuscipes populations in Uganda. Parasit Vectors.

[31] Gillham NW (1994). Organelle genes and genomes.

[32] Rokas A, Williams BL, King N, Carroll SB (2003). Genome-scale approaches to resolving incongruence in molecular phylogenies. Nature.

[33] Whittaker JC, Harbord RM, Boxall N, Mackay I, Dawson G, Sibly RM (2003). Likelihood-based estimation of microsatellite mutation rates. Genetics.

[34] Mishmar D, Ruiz-Pesini E, Golik P, Macaulay V, Clark AG, Hosseini S, Brandon M, Easley K, Chen E, Brown MD, Sukernik RI, Olckers A, Wallace DC (2003). Natural selection shaped regional mtDNA variation in humans. Proc Natl Acad Sci U S A.

[35] Challier A, Laveissière C (1973). Un nouveau piège pour la capture des glossines (Glossina: Diptera, Muscidae): description et essais sur le terrain. Cah ORSTOMSérie Entomol Médicale Parasitol.

[36] Echodu R, Sistrom M, Hyseni C, Enyaru J, Okedi L, Aksoy S, Caccone A. Genetically distinct Glossina fuscipes fuscipes populations in the lake Kyoga region of Uganda and its relevance for human African trypanosomiasis. Biomed Res Int. 2013. doi:10.1155/2013/614721 10.1155/2013/614721 PMC3807537 24199195

[37] Rozas J, Sanchez-DelBarrio JC, Messeguer X, Rozas R (2003). DnaSP, DNA polymorphism analyses by the coalescent and other methods. Bioinformatics.

[38] Excoffier L, Lischer H. Arlequin 3.5: An Integrated Software Package for Population Genetics Data Analysis. 2011. doi:10.1111/j.1755-0998.2010.02847.x PMC2658868 19325852

[39] Weiss KM (1991). Genetic data analysis: Method for discrete population genetic data. By B. S. Weir. xii + 337 pp. Sunderland, MA: Sinauer associates, 1990, $27.00 (paper), $48.00 (cloth). Am J Hum Biol.

[40] Excoffier L, Smouse P, Quattro J (1992). Analysis of molecular variance infered from metric distances among DNA haplotypes: application to human mitochondrial DNA restricyion data. Genetics.

[41] Bandelt HJ, Forster P, Rohl A (1999). Median-joining networks for inferring intraspecific phylogenies. Mol Biol Evol.

[42] David Posadaand KeithA. Crandall (2001) Intraspecific gene genealogies: trees grafting into networks. In: TRENDS Ecol. Evol. Vol.16 No.1 January 2001. 10.1016/s0169-5347(00)02026-7 11146143

[43] Parks DH, Porter M, Churcher S, Wang S, Blouin C, Whalley J (2009). GenGIS: A geospatial information system for genomic data. Genome Res.

[44] Corander J, Sirén J, Arjas E (2008). Bayesian spatial modeling of genetic population structure. Comput Stat.

[45] Corander J, Corander J, Marttinen P, Marttinen P, Tang J, Tang J. BAPS: Bayesian Analysis of Population Structure. Analysis. 2007;1–28.

[46] Smouse PE, Long JC, Sokal RR (1986). Multiple Regression and Correlation Extensions of the Mantel Test of Matrix Correspondence. Syst Zool.

[47] Peakall R, Smouse PE (2006). GENALEX 6: Genetic analysis in Excel. Population genetic software for teaching and research. Mol Ecol Notes.

[48] Harpending HC. Signature of ancient population growth in a low-resolution mitochondrial DNA mismatch distribution. Hum. Biol. an Int. Rec. Res. Hum Biol. 1994;66-591-600. 8088750

[49] Tajima F (1989). Statistical method for testing the neutral mutation hypothesis by DNA polymorphism. Genetics.

[50] Fu YX (1997). Statistical Tests of Neutrality of Mutations Against Population Growth, Hitchhiking and Background Selection. Genetics.

[51] Kvie KS, Hogner S, Aarvik L, Lifjeld JT, Johnsen A (2013). Deep sympatric mtDNA divergence in the autumnal moth (Epirrita autumnata). Ecol Evol.

[52] Hurst GDD, Jiggins FM (2005). Problems with mitochondrial DNA as a marker in population, phylogeographic and phylogenetic studies: the effects of inherited symbionts. Proc Biol Sci.

[53] Richly E, Leister D (2004). NUMTs in sequenced eukaryotic genomes. Mol Biol Evol.

[54] Abila PP, Slotman MA, Parmakelis A, Dion KB, Robinson AS, Muwanika VB, Enyaru JC, Okedi LM, Aksoy S, Caccone A (2008). High levels of genetic differentiation between Ugandan Glossina fuscipes fuscipes populations separated by Lake Kyoga. PLoS Negl Trop Dis.

[55] Beadell JS, Hyseni C, Abila PP, Azabo R, Enyaru JCK, Ouma JO (2010). Phylogeography and population structure of Glossina fuscipes fuscipes in Uganda: implications for control of tsetse. PLoS Negl Trop Dis.

[56] Bouyer J, Balenghien T, Ravel S, Vial L, SidibÉ I, ThÉvenon S, Solano P, De MeeÛs T. Population sizes and dispersal pattern of tsetse flies: Rolling on the river? Mol Ecol. 2009. doi:10.1111/j.1365-294X.2009.04233.x 10.1111/j.1365-294X.2009.04233.x 19457176

[57] Buburuzan L, Gorgan L, Bara I. Types of Dna Used in Speciation and Phylogeny Studies. Analele Ştiinţifice ale Universităţii "Alexandru Ioan Cuza”, Secţiunea Genetică şi Biologie Moleculară, TOM VIII. 2007.

[58] Dyer RJ, Nason JD, Garrick RC (2010). Landscape modelling of gene flow: improved power using conditional genetic distance derived from the topology of population networks. Mol Ecol.

[59] Albert M, Wardrop NA, Atkinson PM, Torr SJ, Welburn SC. Tsetse Fly (G.f. fuscipes) Distribution in the Lake Victoria Basin of Uganda. (2015). PLoS Negl Trop Dis. 2015; 9(4):e0003705. doi:10.1371/journal.pntd.0003705. 10.1371/journal.pntd.0003705 PMC4398481 25875201

