# Supplementary figures and images for: Mitochondrial DNA sequence divergence and diversity of Glossina fuscipes fuscipes in the Lake Victoria basin of Uganda: implications for control
Source: Parasit Vectors. 2015 Jul 22;8:385. doi: 10.1186/s13071-015-0984-1 (PMC4511262; doi:10.1186/s13071-015-0984-1)

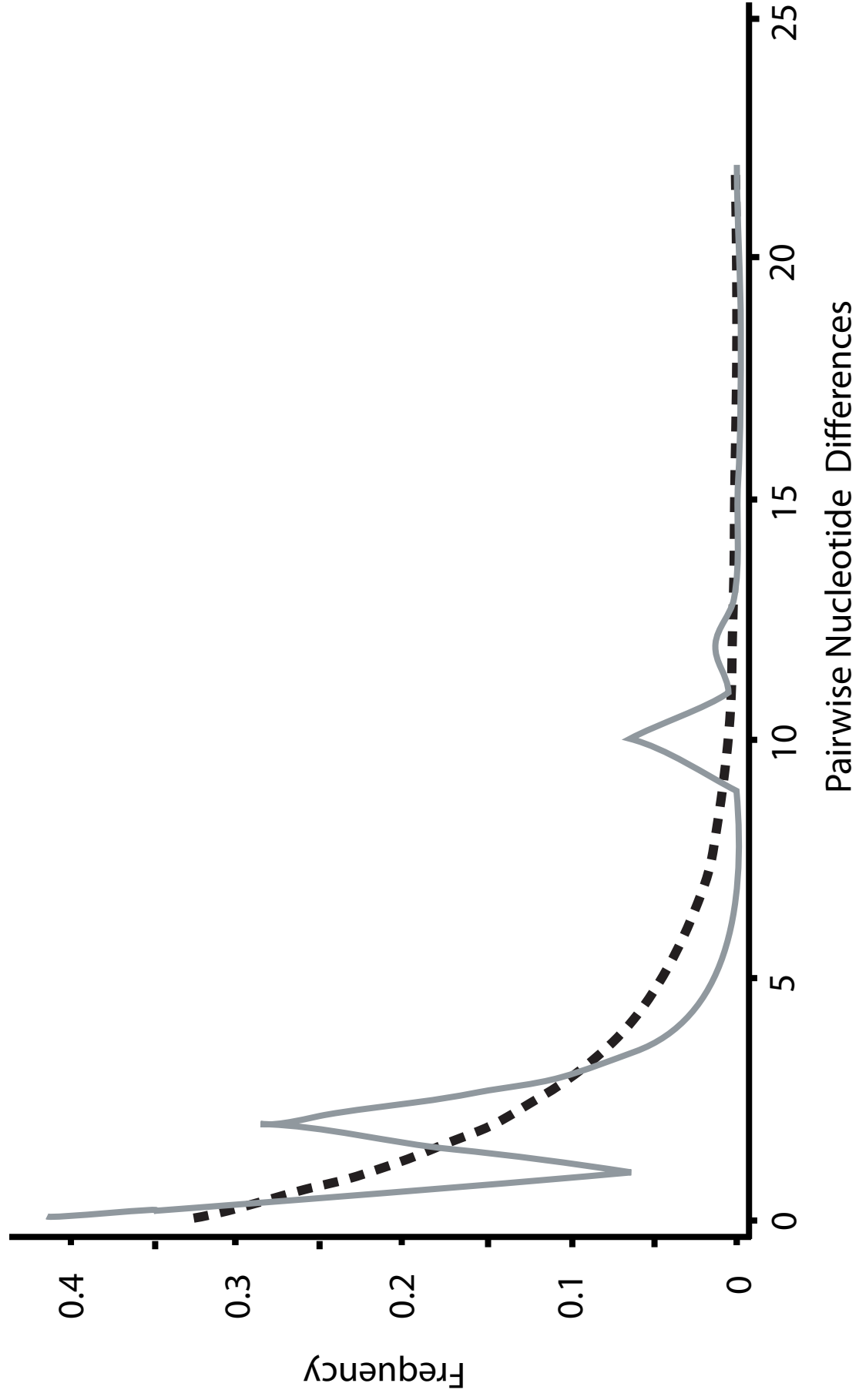

Supplement: Additional file 1: Figure S1. — Mismatch distributions plot [48] obtained using pairwise differences in mitochondrial COII sequence nucleotides for Glossina f. fuscipes in the lake Victoria Basin, Uganda. On the X-axis are the pairwise nucleotide differences, Y-axis are the number of pairs (Frequency). The solid grey lines show observed frequency distribution while the dotted black lines show the distribution expected under constant growth. The data were obtained using DNASP version 5.10 [37]. [file 13071_2015_984_MOESM1_ESM.pdf]

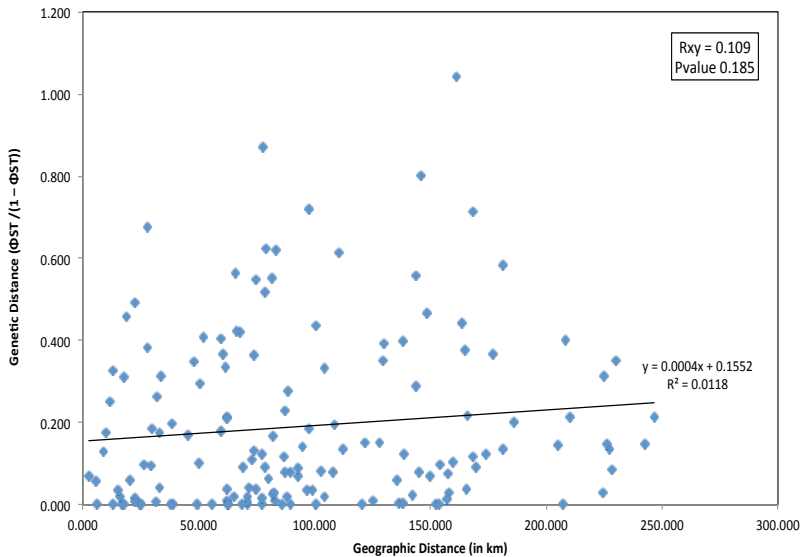

Supplement: Additional file 2: Figure S2. — Mantel Test plot of genetic distance (Φst /(1- Φst)) versus geographic distance for pairwise comparisons among 18 localities of G. f. fuscipes in the lake Victoria Basin, Uganda. Blue dots represent pairwise comparisons of localities and the black line is the linear correlation of genetic and geographic distances across the basin. There is no isolation by distance (R = 0.109, P value = 0.185). [file 13071_2015_984_MOESM2_ESM.pdf]
